# A Proposal for a Solar Position Sensor System with Multifiber Optical Cable

**DOI:** 10.3390/s24113269

**Published:** 2024-05-21

**Authors:** Fernanda Oliveira, Gustavo Cruz, Maria Barbosa, Fernando Junior, Ricardo Lima, Luis Gómez-Malagón

**Affiliations:** 1 Departament of Electrical Energy Systems, University of Technology Sydney, 15 Broadway, Ultimo, NSW 2007, Australia; fernandaatavaress@gmail.com; 2 Postgraduate Program in Systems Engineering, Polytechnic School of Pernambuco, University of Pernambuco, Recife 50720-001, PE, Brazil; gpcc@poli.br (G.C.); lagomezma@poli.br (L.G.-M.); 3 Polytechnic School of Pernambuco, Recife 50720-001, PE, Brazil; brmksb@poli.br (M.B.); fnsj@poli.br (F.J.)

**Keywords:** optical fiber sensor, concentrator photovoltaic, solar tracker, electronic instrumentation

## Abstract

A solar position sensor is an essential optoelectronic device used to monitor the sun’s position in solar tracking systems. In closed-loop systems, this sensor is responsible for providing feedback signals to the control system, allowing motor adjustments to optimize the angle of incidence and minimize positioning errors. The accuracy required for solar tracking systems varies depending on the specific photovoltaic concentration. In the case of the concentrator photovoltaic (CPV), it is normally essential to track the sun with a position error of less than ±0.6°. To achieve such precision, a proposed sensor configuration composed of low-cost embedded electronics and multifiber optical cable is subjected to characterization through a series of measurements covering range, sensitivity, and resolution. These measurements are performed in controlled indoor environments as well as outdoor conditions. The results obtained exhibit a resolution of $2.6\times 10^{-3}$ degrees when the sensor is illuminated within its designated field of view of ±0.1°, particularly in external conditions. Considering the performance demonstrated by the proposed solar position sensor, coupled with its straightforward modeling and assembly compared to position sensors documented in the literature, it emerges as a promising candidate for integration into solar tracking systems.

## 1. Introduction

The solar tracking system can follow the sun using different approaches: open-loop, closed-loop, and hybrid-loop [1]. These strategies have the objective to minimize the incident angle [2]. It can be achieved by an open-loop solar tracking strategy using the Solar Position Algorithm (SPA), which is based on the geometrical relations between the sun and the earth. Another alternative is the closed-loop strategy, which uses the sun position sensor signal as a feedback in a closed-loop control network. This strategy usually works well under direct normal irradiance. However, under cloudy sky conditions or at sunrise and sunset conditions, the sensors do not work properly and hybrid strategies are employed. It means that, for example, under clear and cloudy sky conditions, the closed-loop and open-loop strategies are used, respectively. For closed-loop and hybrid-loop strategies, the sun position sensor is an important component to guarantee the precision of the solar tracking system. The resolution of the sun position sensor depends on the specific solar technology being used, which can be categorized into systems with or without concentration. In the case of solar systems without concentration, a resolution of a few degrees is generally considered acceptable. For example, for a single axis polar mount tracking system, the energy output losses are less than 1% considering a constant tracking error of 6.92% [3].

Concentrator photovoltaic (CPV) systems have a specific requirement known as the acceptance angle, which is defined as the maximum angle of Direct Normal Irradiation (DNI) incidence that can be intercepted by the solar concentrator [4], or the off-tracking angle at which the power output drops below 90%, and its value is typically around ±0.6° [5]. To fulfill this requirement, a solar tracker system must incorporate a solar position sensor capable of measuring angles with a resolution lower than the acceptance angle [6]. Moreover, it is crucial to ensure the long-term stability and durability of the solar tracking system. The precision and accuracy of the solar position sensor must be maintained consistently over several years to guarantee reliable tracking performance. This may involve selecting high-quality components, implementing robust calibration and maintenance procedures, and accounting for environmental factors that can affect the sensor’s performance over time.

Reviews about solar tracking systems involve different aspects such as closed-loop and open-loop types of sun tracking systems [7] and the best tracking methods to achieve the best performance [8]. For example, the common design explored in the literature is a Manual/Automatic Hybrid Dual Axis Solar Tracking System that tracks the sun’s daily and seasonal motions, and is capable of improving the overall energy efficiency by around 35%, over a fixed installation [9]. Typically, two-axis solar tracking systems are based on geometric and astronomical equations and artificial intelligence; however, these techniques are expensive and other approaches are explored, for example, based on analog circuits using the Wheatstone bridge circuit [10].

One common sun position sensor utilizes a pair or multiple Light-Dependent Resistors (LDRs) positioned with an obstacle in between. When the light source is directly overhead, the LDRs register identical signals. However, as the angle of incidence deviates from zero degrees, the presence of a shadow alters the signals detected by the LDRs, providing information about the position of the light source [8]. The use of LDRs as a sun position sensor is employed in Parabolic Trough Collector (PTC) [11] and photovoltaic panels [12]. A variation of this setup consists of placing an optical fiber in the position of the LDRs to collect the light, and to receive the collected light by LDRs at the end of the optical fiber [13].

Another sensor is based on a four-quadrant configuration [8], which is mounted on a tracking plane to enhance the sensitivity of photocurrent measurements. For this application, the whole sensor consists of a pipe with a small hole or slit located at one end to spatially filter the light that strikes the four-quadrant sensor located at the other end of the pipe. The error sources of this sensor include assembly error, light spot distortion, and signal characteristics of electronic devices, which make the calibration of the sun sensor cumbersome and limit the accuracy. To overcome these problems, deep neural networks [14] and numerical analysis [15] techniques are employed.

Additionally, the Charge-Coupled Device (CCD) is explored for sensing the sun position. This technique is employed for the solar observation satellite [14]. Under radiation, the temperature of the sensor can affect the phase of the CCD sampling signal and compensation must be taken into account [16]. Also, the analysis of the image is proposed to improve accuracy using sub-pixel interpolation [17].

Various options exist for optical position sensors, with many of them employing detectors positioned in front of solar radiation, featuring exposed areas larger than one hundred square millimeters [18]. However, these sensors have certain drawbacks. Temperature changes and environmental disturbances can introduce variations in the sensor response, primarily caused by semiconductor behavior alterations and electronic instrumentation exposure. An alternative approach involves the utilization of optical fibers to capture solar radiation and transmit it to a processing module located away from the tracking system. This light collection technique is commonly employed in daylighting control systems.This approach is explored in the literature mainly as an alternative to reduce the lighting load, improving the efficiency of artificial lighting [19]. These systems can use plastic/silica fibers and use different light collection systems [20]. For example, parabolic mirror and fresnel lens are available for fiber-based daylighting systems [21]. Additionally, photovoltaic cells can be coupled with this system in a hybrid system [22]. Other applications include photocatalysis for treatment of textile wastewater [23] and hydrogen production [24]. These examples show that there is an effort to use optical fiber for environmental applications [25].

As exposed previously, solar concentrators and fresnel lenses are frequently used to focus sunlight beams onto the optical fibers. This configuration can be used for sun position sensors; however, few studies have explored this potential. For example, multifibers were used, placing one end in a cross-shaped structure, and the other end was used to collect the signal by photodiodes. Results indicated that the tracking error was 0.12° and its viewing angle was more than ±60° [26]. Another alternative explored the directionality of light coupling to a waveguide [27]. As presented, optical fibers have potential to be used in solar applications such as sun position sensors [26], torsion sensors of photovoltaic structures [28], and also daylighting and wastewater treatment [23]. However, special attention must be paid to the UV degradation of the optical fiber and also to the peak of the spectral responsivity of the sensor. In the case of sun position sensors, the use of optical filters reduces the effect of diffuse radiation and increases the performance of sun tracking systems regardless of the type of controller used in the solar tracker [29].

Given the aforementioned context, this article aims to contribute to the daylighting system research by presenting a concise overview of a solar position sensor’s straightforward topology, boasting a resolution of $8.6\times 10^{-4}$ and $2.6\times 10^{-3}$ degrees for indoor and outdoor experiments, respectively. Section 2 elucidates the functioning of the optical component of the sensor system, including a simulation depicting its response to a monochromatic and collimated beam. Subsequently, in Section 3, a detailed exposition of the optical components is provided, featuring an optical cable bundle housing seven optical fibers, a converging lens, and photodetectors. Additionally, the sensor system incorporates a processing module equipped with circuits for signal conditioning from the photodetectors, alongside a microcontroller platform, facilitating automated data acquisition and rotation platform control. The ensuing Section 4 showcases the outcomes of both indoor and outdoor experiments, characterizing the device’s range, resolution, and sensitivity. Ultimately, the concluding remarks affirm that the obtained results for resolution (0.001° and 0.003° for indoor and outdoor conditions, respectively), surpass those documented in the existing literature on daylighting systems based in optical fibers, which reports position accuracy of 0.1°. As predicted, the arrangements of fibers closely improved the spatial resolution [17].

## 2. Operation Principle

**Figure 1:**
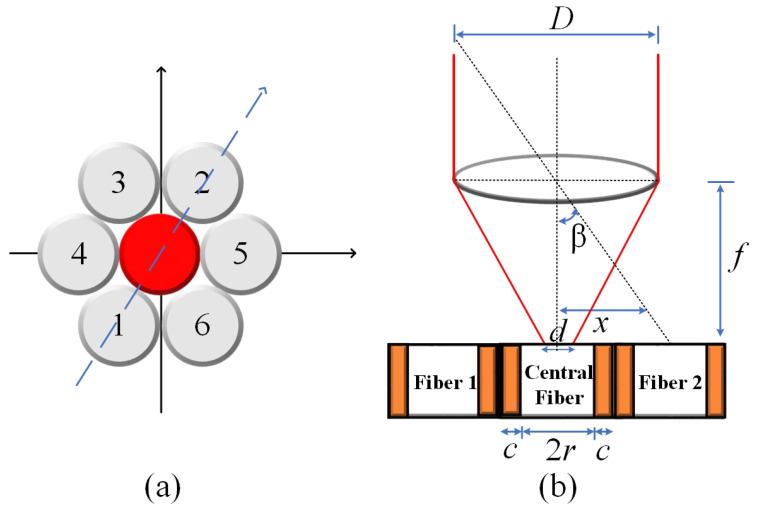
(**a**) Drawing of optical fiber arrangement in Thorlabs^®^ UM22-100-FBUNDLE cable (Thorlabs, Newton, NJ, USA). The central fiber is highlighted in red and the arrow passing by fiber1–central fiber–fiber 2 is the direction employed for the optical characterization. (**b**) Geometry of the system.

The optical fibers within the optical cable are depicted in Figure 1a. This configuration reveals the presence of a central fiber accompanied by six neighboring fibers. To investigate the optical response of the fiber optic sensor, it is subjected to illumination along the path illustrated in Figure 1a. In this analysis, a single degree of freedom is considered to assess the tracking position. Figure 1b presents the proposed model aimed at elucidating the characteristics of the optical sensor when exposed to a collimated light beam with wavelength λ, directed through a converging lens. In the model, β is the incident angle, *c* is the sum of cladding and coating thickness, *r* is the radius of the optical fiber, *D* is the diameter of the beam in the plane of the lens, *d* is the focalized beam diameter, and *f* is the focal length of the lens.

From the geometry, the fibers are fully illuminated if the position of the spot of the focalized light, *x*, satisfies the following conditions:

(1) r−d/2>x>0.

For the lateral fibers 1 and 2:

(2) 3r+2c−d/2>x>r+2c+d/2.

where the diameter of the focalized beam, *d*, is approximately given by [29,30]:

(3) $$d=\frac{4\lambda f}{\pi D}.$$

The central fiber is fully illuminated if the incident angle is on the field of view (FOV). It means that for β=±θ, the incident angle is given by [31,32]:

(4) $$\theta =\left(\frac{r-d/2}{f}\right).$$

**Figure 2:**
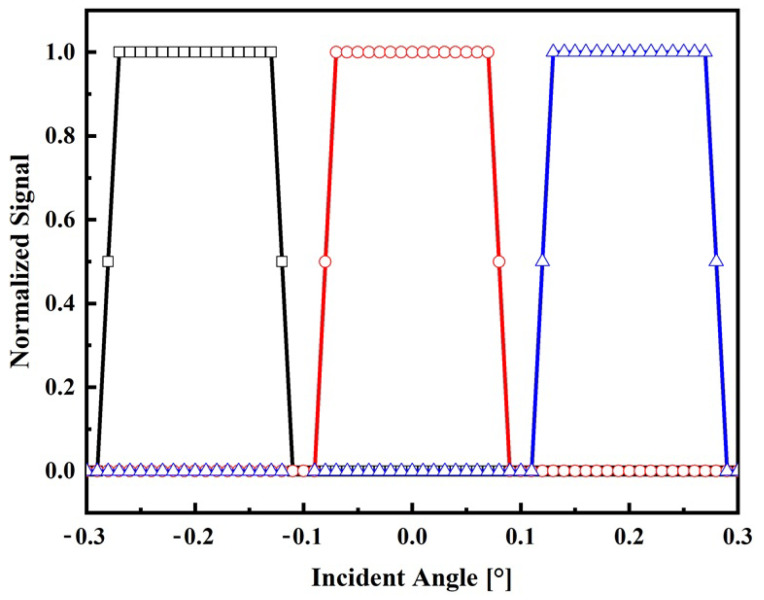
Theoretical response of the sensor on the angle of the incident radiation. Normalized signal of the lateral fiber 1 (black square), central fiber (red circle), and lateral fiber 2 (blue triangle).

By examining the geometry depicted in Figure 1b and taking into account the specific values of *d* = 14 µm, *D* = 2 mm, and *f* = 35 mm, the incident angle for the central fiber was calculated using Equation (4). The resulting value is determined to be $\beta =0.07^{\circ }$. Consequently, it implies that the maximum normalized electrical output signal, obtained from the photodetector, is observed within the field of view (FOV) encompassing ±θ. Using Mathcad^®^14, the behavior of the normalized electrical signal is verified, considering that the illuminated fibers are indicated in the path shown in Figure 1a, for angular variation of beam incidence of ±0.3°. The result is shown in Figure 2.

According to the model, it becomes evident that when the incident angles surpass the FOV of the central fiber, the sensor becomes capable of detecting variations by monitoring the output signals of the lateral fibers. Conversely, when the incident angle is around the FOV, the signal remains constant. In practical terms, this implies that the sun tracker system must possess the capability to transmit a control signal to the actuators, enabling them to position the solar surface in a manner perpendicular to the direction of the incident light. In other words, results shown in Figure 2 reveal that if the central fiber is completely illuminated, the incidence angle is less than 0.1°. In contrast, if any other fiber is illuminated, it means that the signal is off-axis and a control procedure must be adopted to illuminate the central fiber. Due to the nature of the sensor, the suggested technique to control the solar tracker using this type of sensor should be based on fuzzy logic rules [33].

## 3. Materials and Methods

**Figure 3:**
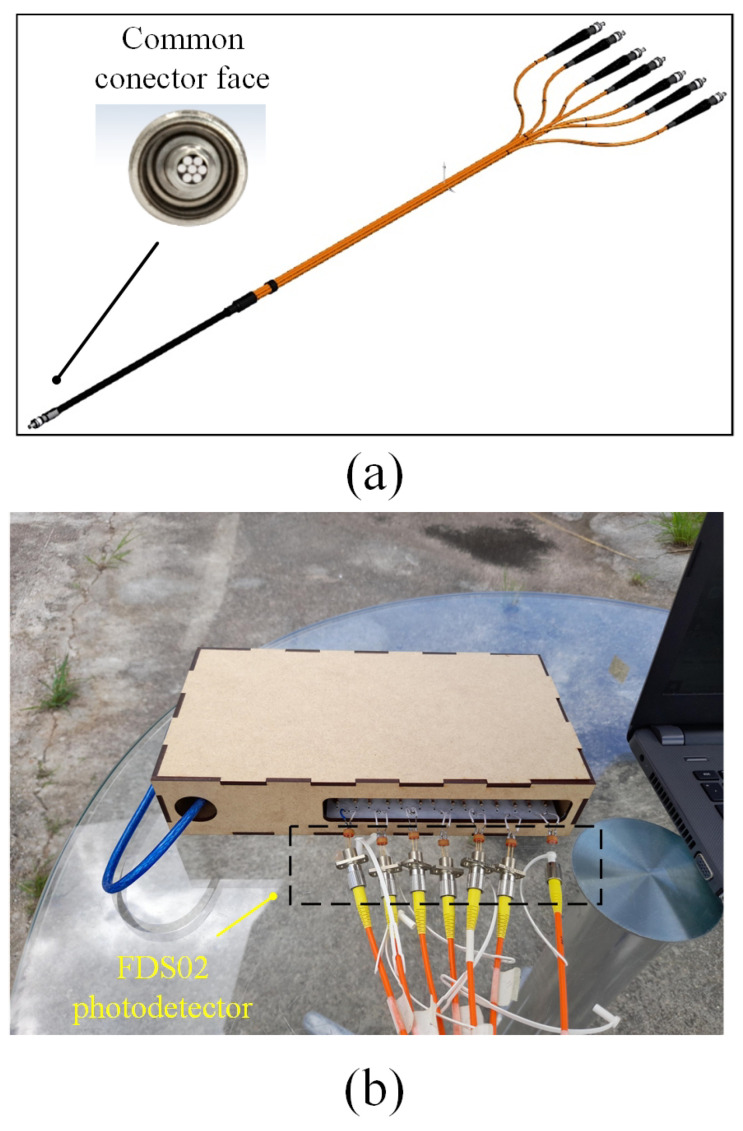
(**a**) Thorlabs^®^ UM22-100-109 FBUNDLE optical cable drawing (Thorlabs, Newton, NJ, USA). (**b**) Thorlabs^®^ FDS02 photodetector (Thorlabs, Newton, NJ, USA) connected to the optical cable.

The optical arrangement of the solar position sensor utilizes a Thorlabs UM22-100-FBUNDLE optical cable, as illustrated in Figure 3a, which comprises seven optical fibers. Each fiber has core/clad diameter of 100 ± 3 μm /110 ± 3 μm and NA = 0.22 ± 0.02. One end of the cable features a common FC-PC connector, while the other end consists of seven individual FC-PC connectors. The optical signal emitted from each fiber optic output is then connected to a Thorlabs^®^ model FDS02 photodetector (Thorlabs, Newton, NJ, USA), as can be seen in Figure 3b. It is highlighted in Figure 3b that one of the fibers is not used because one of the photodectors was defective. Additionally, a biconvex lens with a focal length of 35 mm Model LB1811-ML, Thorlabs^®^ (Thorlabs, Newton, NJ, USA) and an opening diameter of 2 mm is integrated into the optical arrangement. The primary function of this lens is to focus the radiation individually onto each fiber, ensuring precise and targeted illumination.

**Figure 4:**
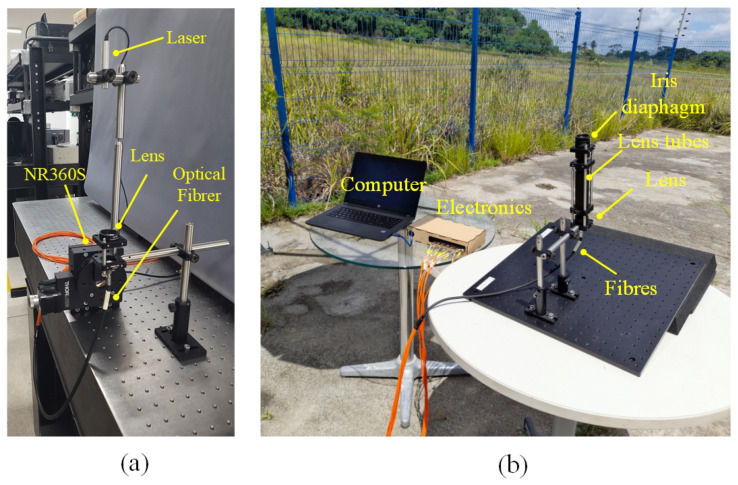
Experiment setup: (**a**) indoor; (**b**) outdoor.

Measurements at indoor conditions are performed in a room at 25 °C. A laser with a wavelength of λ = 635 nm is mounted on a motorized rotation stage (NR360S Thorlabs^®^ (Thorlabs, Newton, NJ, USA) to emulate the sun’s motion controlling precisely the direction of the light source, as depicted in Figure 4a. This configuration allows for achieving a beam waist of approximately 14 μm at the focal plane. During the measurements, the light collected by the fibers is recorded at intervals of 0.027°. This spacing is determined by dividing the smallest motor step (1.8°) by the gear ratio of the mechanical transmission (66:1). The trajectory of the light is illustrated in Figure 1a, providing a visual representation of its path through the optical setup.

The outdoor measurements are conducted under clear sky conditions and the external temperature is about 30 °C. In this experimental setup, the optical cable and lens are securely mounted on a platform, as depicted in Figure 4b. The platform is tilted at an angle aligned with the trajectory of the sun, following the trace illustrated in Figure 1a.

To enhance the optical performance and ensure selective collection of direct radiation while avoiding the capture of reflected light from the surroundings, a lens tube equipped with an iris diaphragm is incorporated into the setup. During the outdoor measurements, variations in the incident angle are considered at increments of 0.02°. The measurements are recorded at a sample rate of 4.8 s, taking into account the Earth’s angular speed around the sun, which is approximately 15° per hour.

**Figure 5:**
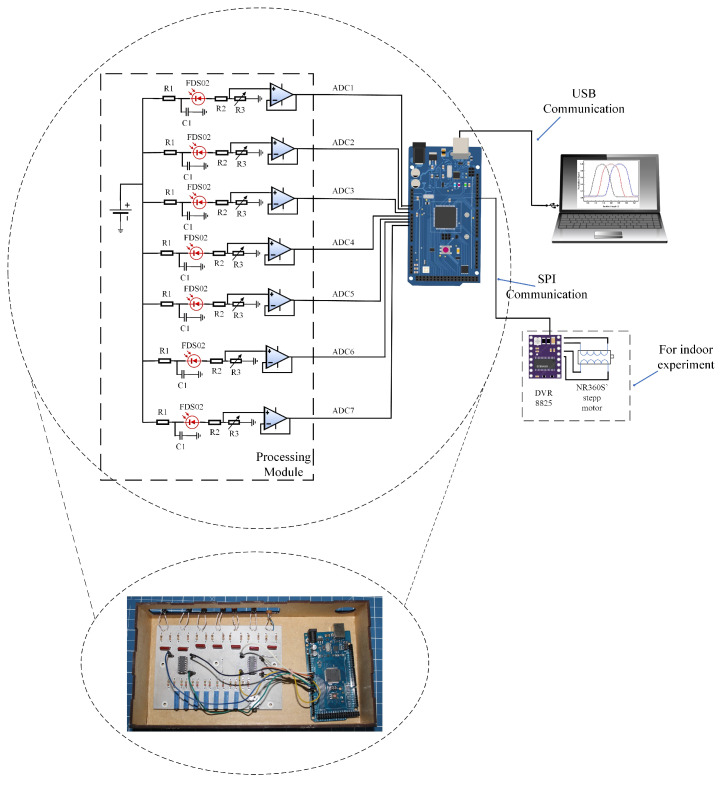
Photograph and block diagram of the electronic instrumentation for processing the optical signal of the optical cable. The DVR8825 module stands out for indoor experiments.

Figure 5 illustrates the block diagram of the electronic instrumentation employed for the experiments involving the optical fiber sensor, utilizing a processing module in conjunction with the Arduino Mega^®^ microcontroller platform and the photograph of the printed circuit board made, as well as the Arduino Mega. The processing module consists of seven current/voltage converters designed to transform the photocurrent generated by the FDS02 photodetector into a corresponding voltage signal. To mitigate potential noise interference in the polarization voltage of the photodiode, each converter incorporates an RC circuit configured as a low-pass filter at its input.

To ensure the voltage values at the output of the photodiode are normalized, an adjustable voltage divider is employed. Furthermore, a buffer-type voltage follower circuit is implemented to facilitate proper impedance coupling between the circuitry and the Analog-to-Digital Converter (ADC) input of the Arduino, thereby optimizing signal integrity and accuracy.

To automate the Thorlabs^®^ NR360S motor (Thorlabs, Newton, NJ, USA) for indoor experiments, the inclusion of a stepper motor driver DRV8825, Pololu^®^ (Pololu, Las Vegas, NV, USA) is necessary. The Arduino Mega’s resident program allows the user to define the desired range of values for the angular displacement (β) and the corresponding steps. This information is then transmitted via the SPI bus from the Arduino to the DRV8825, ensuring the accurate activation and control of the NR360S’s stepper motor. For both indoor and outdoor conditions, during each variation of the angular position of the UM22-100-FBUNDLE, the program acquires the voltage signal from the processing module (ADC1 to ADC7). The acquired data are then stored in .txt format, facilitating further post-processing and analysis on a notebook.

## 4. Results and Discussion

**Figure 6:**
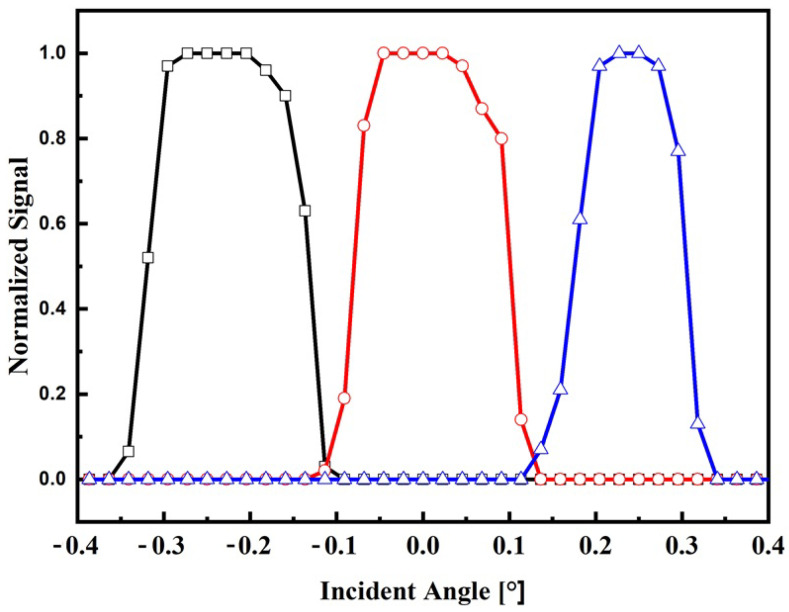
Electrical response on the incident angle for indoor conditions. Normalized signal of the lateral fiber 1 (black square), central fiber (red circle), and lateral fiber 2 (blue triangle).

**Figure 7:**
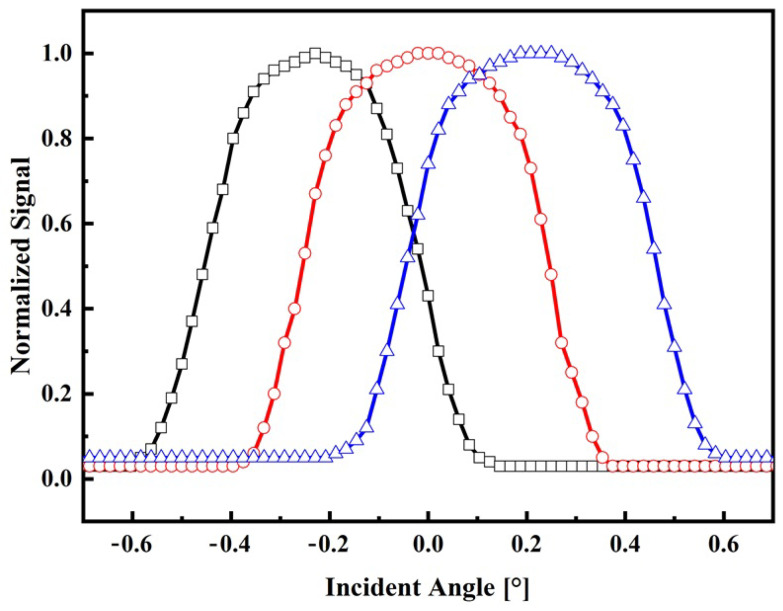
Electrical response on the incident angle for outdoor conditions. Normalized signal of the lateral fiber 1 (black square), central fiber (red circle), and lateral fiber 2 (blue triangle).

In this section, the sensor system is characterized through a series of indoor and outdoor experiments utilizing the devices outlined in the materials and methods section. The objective is to evaluate the normalized signal of the sensor output as a function of the angle of incidence for both experimental setups. Based on the obtained results, the range, resolution, and sensitivity of the sensor system are determined for both indoor and outdoor conditions. These parameters provide crucial insights into the performance and capabilities of the sensor system in each respective situation. The normalized output signals for indoor and outdoor conditions are shown in Figure 6 and Figure 7, respectively.

By examining Figure 6, it becomes apparent that the electric response of each fiber does not overlap as initially predicted by the theoretical model presented in Figure 2. Additionally, the mounted sensor exhibits a field of view (FOV) of approximately ±0.1°, aligning with the desired specifications for concentrator photovoltaic (CPV) applications. Furthermore, it is evident that only the central fiber is illuminated when the incident angle remains within the range of ±0.1°. However, for incident angles exceeding this threshold, the lateral fibers become illuminated, indicating that the solar surface has deviated from its central position. This observation serves as a practical indication of the sensor’s ability to detect and monitor the misalignment of the solar surface in relation to the desired central position.

During the operation of the sensor under clear sky conditions (1000 W/m^2^), as depicted in Figure 7, it is observed that the electrical signal profile deviates from the theoretical model. These differences can be attributed to the spectral profile of the sun’s radiation, which is not uniformly focused by the lens due to chromatic aberration [29]. As a result of this phenomenon, when the incident angle is 0°, the central fiber receives the majority of the radiation, while the lateral fibers are equally illuminated. When the incident angle reaches ±0.15°, two fibers are equally illuminated, while the remaining fiber remains in darkness. This distinct pattern of illumination provides valuable information for determining the position of the sun through signal processing techniques. Also, from Figure 7, it is observed that the minimum irradiance that can be detected by the sensor is about 5% (50 W/m^2^) of its maximum without compromising the signal–noise ratio. This low level of irradiance is still lower than irradiance commonly considered for diffuse radiation, which is about 10–15% of the total global hemispherical irradiance [34].

The characterization of the sensor involves determining its range, sensitivity, and resolution. In the case of the proposed sensor, the range refers to the maximum and minimum values of the incident angle that can be effectively measured [35]. The sensitivity, on the other hand, is the ratio between the electrical output signal (Δ*V*), given in volts, and the corresponding change in the incident angle outside the core fiber (Δβ), expressed as sensitivity = ΔV/Δβ [35].

To calculate the resolution, a 10-bit analog-to-digital (A/D) converter is considered, which accepts input values in the range of 0–5 V. As a result, the minimum voltage that can be reliably measured is approximately 4.9 mV (calculated as 5 V/1023). Alternatively, the resolution can be expressed as 4.9 mV divided by the sensitivity.

In summary, the range indicates the span of incident angles that can be measured, the sensitivity quantifies the relationship between the electrical output signal and the incident angle, and the resolution represents the smallest detectable change in the incident angle that the sensor can reliably monitor.

In addition to the above considerations, the range of the sensor is determined by identifying the maximum and minimum values of incident angles that can be detected [35]. Since the electrical signal exhibits minimal variation within the field of view (FOV), the sensitivity and resolution are calculated specifically at the interface between the optical fibers. The corresponding values are summarized in Table 1.

In order to compare with other technologies, some examples are given in Table 2 following the classification given in the literature [17]. According to it, sun position sensors can be classified as (a) collimated sun sensor, (b) sun-pointing sensor, (c) tilted mount photo sensor, and (d) hybrid sensors.

As reported in Table 2, the collimating sensor show the lowest error tracking among the other architectures. It is verified in outdoor conditions using a four-quadrant sensor [36]. Then, the use of this concept in miniaturized devices is explored in the literature. For example, a CMOS image sensor is designed as a sun sensor for microsatellite application with resolution 0.04° in ±50° FOV [37]. Other recent sun position sensors compatible with CMOS technology have reported accuracies of 5.7° in a ±37° FOV [38] and 0.6° in a ±26° FOV [39]. Commercially, the Sum Mems [40] manufactures sun sensors with angle resolution of 0.001° and FOV of 10°. Then, comparing the proposed sun position sensor with the micro digital sun sensor technology, the resolutions are similar (0.001°), and the ratio of FOVs is at least one order of magnitude. However, the proposed sensor is an optical device that is not affected by electromagnetic interference. Ultimately, the concluding remarks affirm that the obtained results for resolution (0.001° and 0.003° for indoor and outdoor conditions, respectively) surpass those documented in the existing literature on daylighting systems, which reports position accuracy of 0.1°. As predicted, the arrangements of fibers closely improved the spatial resolution [41]. Thus, the proposed sensor system emerges as a promising candidate for integration into solar tracker systems.

**Table 2:** Comparison of different sun position sensor technologies.

	Description	Solar Tracking Error [°]/Accuracy [°]	Reference
Collimating sensor			
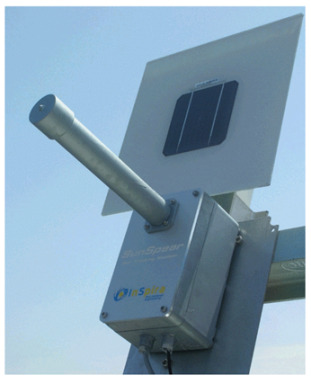	A sunlight collimating pipe placed over the Position-Sensitive Device (PSD) surface.	0.05/	[42]
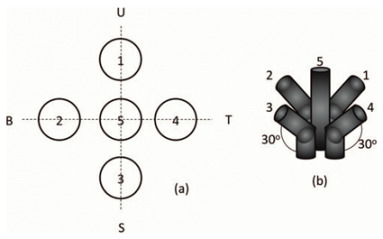	Arrangement of five photodiodes. One photodiode in the middle and four photodiodes bent to 30 degrees.	5/0.1	[43]
Sun-pointing sensor			
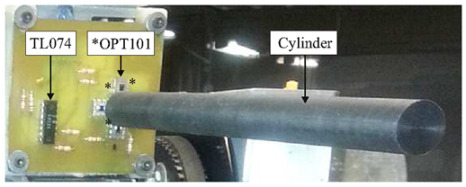	Four-quadrant Light-Dependent Resistors (LDR) and a cylinder	0.134/-	[44]
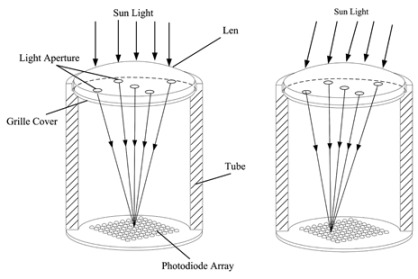	Light is directed through a lens and five 2 mm holes, then focused onto a photodiode matrix to ascertain the sun’s position via computational processing.	0.1/0.1	[41]
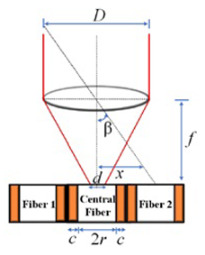	The incident light is focused by a lens (F = 35 mm, D = 25 mm) onto a plane with a 7-multifiber bundle connected to photodiodes.	0.003/-	Present Work
Tilted mount sensor			
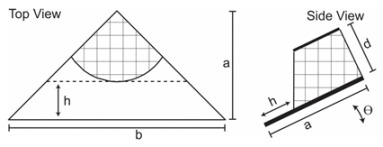	A tilted mount photodetector with four quadrants, each containing a photodetector positioned at a 45°-45°-90° right triangle with dimensions b = 2 mm and a = 1 mm. A 1 mm diameter pinhole permits light entry into the sensor.	1/-	[45]
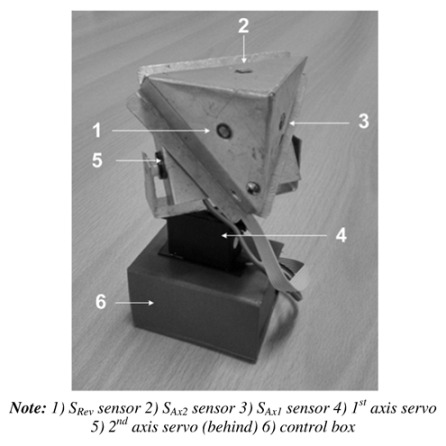	Three identical LDRs placed in parallel and symmetrically to each side of the pyramid.	1.67/-	[46]
Hybrid sensor			
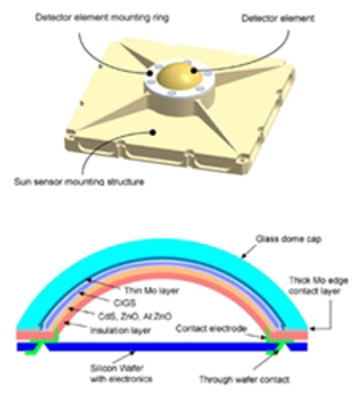	Under the spherical glass dome cap, a CIGS layer is deposited. Under illumination, six electrodes pick up the generated photocurrent by the CIGS layer. The angle of the incident light is determined from the distribution of the photocurrents to the electrodes.	1/-	[47]
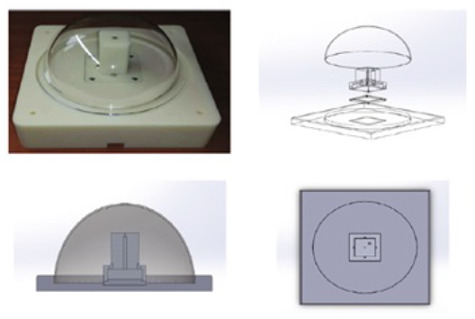	Hybrid solar tracking device that uses four photo-sensors to track the sun when the solar radiation is higher than 400 W/m^2^. Otherwise, a GPS-based program uses an astronomical formula to track the sun.	1/-	[48]
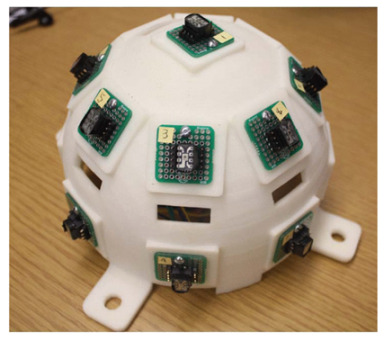	The sun position sensor, employing an 80 mm hemispherical geometry, integrated 17 TSL230R light intensity sensors within its structure, alongside a microcontroller and an AHRS sensor for altitude compensation, operating on the principle of light presence or absence to generate 3D transformation matrices.	5/-	[49]

## 5. Conclusions

The results presented in this article indicate a streamlined optical configuration, contrasting favorably with existing daylighting systems documented in prior research. Moreover, such a setup fulfills the essential criteria for solar trackers utilized within concentrated photovoltaic systems, complemented by cost-effective electronic instrumentation for processing.

Notably, deviations observed in the electrical response due to incident angle variations, particularly in outdoor conditions, can be effectively addressed by incorporating a parabolic mirror. This mirror serves to collect solar radiation and focus it onto the surface of the optical fiber, mitigating the influence of chromatic dispersion. It is worth emphasizing that higher sensitivity values and lower resolution values are inversely correlated with the diameter of the focused beam. By reducing the value of the diameter (*d*), sensitivity can be increased. This adjustment in *d* can be accomplished through the implementation of an appropriate lens configuration.

Due to the nonlinear electrical response of the optical sensor on the incident angle, the optical sensor requires calibration. Consequently, the implementation of this system allows for the generation of a comprehensive database containing precise information regarding the tracking position relative to the sun. By harnessing the power of computational techniques such as fuzzy logic and artificial intelligence, it becomes feasible to attain significantly enhanced control over the positioning of the solar tracking system, facilitating more accurate and efficient sun trackers in CPV systems operations.

## Figures and Tables

**Table 1:** Range, sensitivity, and resolution of the sensor for indoors and outdoors.

	Indoor Condition	Outdoor Condition
Range [°]	±0.35	±0.6
Sensitivity [V/°]	5.7	1.9
Resolution [°]	$8.6\times 10^{-4}$	$2.6\times 10^{-3}$

## Data Availability

The data presented in this study are available on request from the corresponding author.
